# 337. Impact of Anti-tuberculosis Drugs on Mycobacteriophage D29 Infectivity in Mycobacterium smegmatis Host Cells

**DOI:** 10.1093/ofid/ofaf695.120

**Published:** 2026-01-11

**Authors:** Juliano H Ito, Jean Meneguello, Regiane Scodro, Katiany Caleffi-Ferracioli, Rosilene Cardoso

**Affiliations:** Laboratory of Medical Bacteriology, Department of Clinical Analysis and Biomedicine, State University of Maringá, Maringá, Parana, Brazil; Laboratory of Medical Bacteriology, Department of Clinical Analysis and Biomedicine, State University of Maringá, Maringá, Parana, Brazil; Laboratory of Medical Bacteriology, Department of Clinical Analysis and Biomedicine,State University of Maringá, Maringá, Parana, Brazil; Laboratory of Medical Bacteriology, Department of Clinical Analysis and Biomedicine,State University of Maringá, Maringá, Parana, Brazil; Laboratory of Medical Bacteriology, Department of Clinical Analysis and Biomedicine, State University of Maringá, Maringá, Parana, Brazil

## Abstract

**Background:**

Tuberculosis (TB) remains a life-threatening disease, particularly due to the growing antimicrobial resistance. The use of mycobacteriophages with anti-TB drugs has been proposed to overcome the rise of multidrug resistance as an alternative to the use of antimicrobials alone. Although interest in phage-antibiotic interactions is increasing, the impact of antimicrobials on phage infectivity is still not well understood. This study aims to evaluate the interaction between anti-TB drugs and the infectivity of mycobacteriophage D29, using efficiency of plating (EOP) assays against *M. smegmatis* mc²155 host cells.

**Figure:**
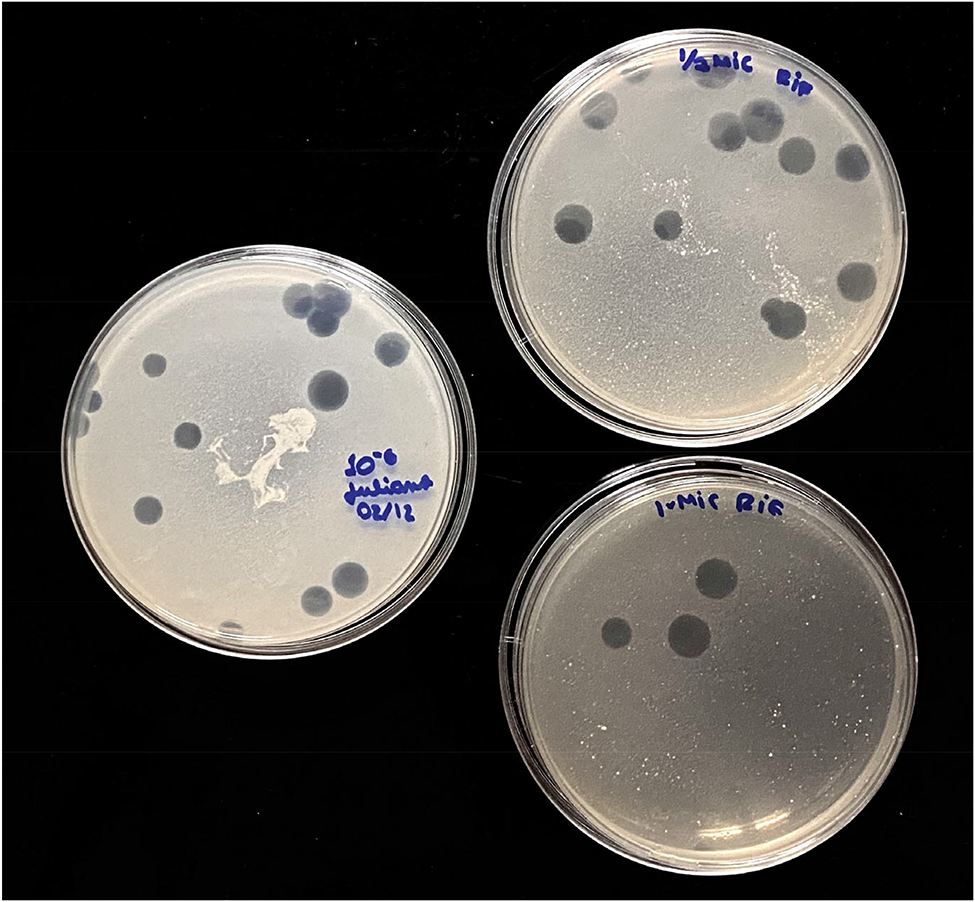
Impact of Anti-TB Drugs on Phage D29 Infectivity: Representative Plaque Assay Plaque formation by mycobacteriophage D29 on Mycobacterium smegmatis mc²155 lawns under different treatment conditions. On the left, the untreated control plate shows a slightly higher plaque count. On the right, two plates with anti-TB drug treatment are shown: the top plate demonstrates moderate plaque reduction, while the bottom plate shows a marked reduction in plaque count. These images highlight the inhibitory effect of anti-TB drug treatment on phage infectivity.

**Figure:**
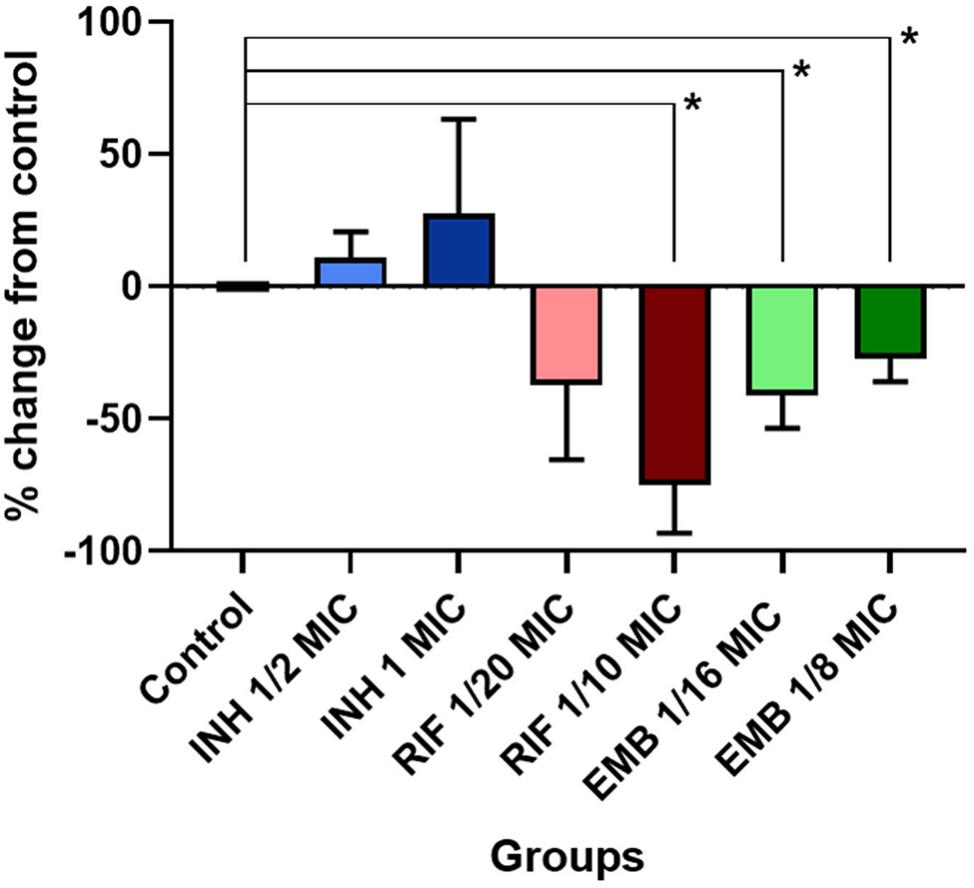
Effect of Anti-TB Drug Concentrations on Mycobacteriophage D29 Infectivity Relative change (%) in plaque-forming units (PFU/mL) of mycobacteriophage D29 compared to untreated control (0%), following exposure to different concentrations of isoniazid (INH), rifampicin (RIF), and ethambutol (EMB) in Mycobacterium smegmatis mc²155. Bars represent mean values, and error bars indicate standard deviation. Asterisks (*) denote statistically significant differences (p < 0.05) compared to the control group.

**Methods:**

*M. smegmatis* was used as a surrogate model for *Mycobacterium tuberculosis.* Phage infectivity under drug-induced stress was assessed by measuring the phage-host interaction through efficiency of plating (EOP) using plaque assays. Experiments were conducted in the presence of subinhibitory concentrations of rifampicin (RIF), isoniazid (INH), and ethambutol (EMB). Due to the inherent variability in plaque-forming unit (PFU) counts across replicates, phage infectivity was expressed as the relative change in PFU/mL (%) compared to the untreated control group, to allow data normalization and enhance statistical analysis.

**Results:**

A statistically significant decrease (p < 0.05) in the relative change of PFU/mL was observed under three conditions compared to the untreated control. Values are expressed as mean ± standard deviation. The reductions in EOP were: 75.04% ± 18.27 (*p*=0.019) at 1/10 MIC of RIF, 41.15% ± 12.52 (*p*=0.047) at 1/16 MIC of EMB, and 27.18% ± 8.70 (*p*=0.047) at 1/8 MIC of EMB. In contrast, INH treatment showed a non-significant increase in EOP with rising INH concentrations, suggesting a potentially distinct interaction mechanism between INH and phage D29.

**Conclusion:**

The results demonstrate that even low concentrations of anti-TB drugs can significantly impact phage infectivity, likely through drug-induced changes in bacterial physiology and phage susceptibility, as evidenced by the significant reduction in EOP. These findings are essential for a better understanding of the complex dynamics between phages and antibiotics, which may contribute to optimizing phage therapy strategies for tuberculosis and other multidrug-resistant infections.

**Disclosures:**

All Authors: No reported disclosures

